# Reconsidering the Imaging Evidence Used to Implicate Prediction Error as the Driving Force behind Learning

**DOI:** 10.3389/fpsyg.2017.01380

**Published:** 2017-08-11

**Authors:** Jiří Čevora, Richard N. Henson

**Affiliations:** MRC Cognition and Brain Sciences Unit, University of Cambridge Cambridge, United Kingdom

**Keywords:** learning, neuroimaging, associative learning, prediction error, Hebbian learning

In this paper, we review the evidence that learning is driven by signaling of Prediction Error [PE] by some neurons. We model associative learning in artificial neural networks using Hebbian (non-PE) learning algorithms to investigate whether the data used to implicate PE in learning can arise without actual PE computation. We conclude that the metabolic demands of synaptic change during Hebbian learning would produce a PE-correlated component in functional magnetic resonance imaging (fMRI), which suggests that the research used to imply PE in learning is currently inconclusive.

There is a considerable body of evidence that PE is computed by dopaminergic neurones in ventral midbrain. Single-cell recordings have shown neurons that are excited by unexpected reward, and depressed by unexpected lack of reward (Schultz et al., 1997). This response implies reward PE computation takes place in the brain; however, it does not imply that the PE signal is utilized during learning, and no single-cell study, to our knowledge, has demonstrated this link to learning. Furthermore, these findings have only been obtained with regard to rewarded behavior, while the majority of learning in humans happens in absence of reward (Tolman, 1932).

These concerns can be potentially addressed in fMRI studies by relating a PE-related component of fMRI to subsequent memory, with or without overt rewards. Unfortunately, most fMRI research has focused simply on replicating the single-cell findings by identifying a correlate of PE in the human brain (e.g., McClure et al., 2003; Abler et al., 2006; D'Ardenne et al., 2008), without assessing its effect on behavior. We are only aware of two fMRI studies that attempted to go beyond the single-cell recording findings by demonstrating an effect of a PE-related component in fMRI on learning (Gläscher et al., 2010; McGuire et al., 2014). Both of these studies identify a component of the fMRI signal that is correlated with trial-by-trial estimates of PE from an assumed learning model, and then link that component to subsequent decision-making.

However, PE-correlated fMRI signal does not necessarily originate from PE computation: the BOLD signal measured by fMRI may relate to metabolic changes that are only indirectly related to neural activity. One of the major factors contributing to the BOLD signal is cellular respiration associated mainly with ATP metabolism (Aubert and Costalat, 2002), which is elicited by a large number of cellular processes. Synaptic plasticity has several components working at different timescales (Collingridge et al., 2004), but there are four notable processes that operate at the timescale of these studies: (a) Synaptic transmission of signal, (b) facilitation, which is an important form of short-term synaptic plasticity (Kandel, 2001), (c) migration of receptors, which is crucial components of long-term potentiation and depression (Collingridge et al., 2004), and (d) fast forms of homeostatic activity, which serve as a form of global synaptic scaling and metaplasticity (Pérez-Otaño and Ehlers, 2005). While synaptic transmission (a) is the main energy expense during signaling (up to 55% of signaling cost, Harris et al., 2012), synaptic plasticity (b–d) can increase signaling efficiency up to hundred-fold (Harris et al., 2012) and therefore be expected to have a significant energy budget. Many of these synaptic processes occur rapidly (Collingridge et al., 2004), and could therefore take place within the same timewindow (resolvable by fMRI) as any neural activity related to PE. Thus while the actual energy consumption of synaptic plasticity is unknown (Harris et al., 2012), we conclude that there is a distinct possibility that it is sufficiently large to contribute to the BOLD response.

The outstanding question for this alternative explanation is why synaptic plasticity would correlate with PE, unless PE were computed and used to update synapses. In what follows, we model synaptic plasticity as the magnitude of Hebbian weight update in associative networks, and demonstrate that this quantity correlates with PE even when the learning algorithm does not compute PE.

We consider a modified Hebbian learning rule that includes weight decay term, also called Oja's rule (Equation 1, Oja, 1982). This learning rule does not use the current state of network (e.g., predictions) to inform learning in any way. The only modification from the classic Hebbian algorithm is that the weights decrease linearly at each time step, which is the minimal modification necessary to obtain stable and biologically plausible learning dynamics. We contrast this variant of Hebbian learning with Widrow-Hoff learning algorithm, also modified to include decay to increase its biological plausibility (e.g., Rumelhart and McClelland, 1988) as shown in Equation (2). The formulation of Widrow-Hoff learning rule used here is essentially Hebbian learning scaled by PE. In these equations, *w*_*ij*_ refers to the weight between unit *i* (representing the cue) and unit *j* (representing the outcome), *a*_*i*_/*a*_*j*_ refer to the activity of unit *i*/*j*, *t*_*j*_ refers to a desired output of unit *j*, 0 ≤ *k* < 1 is the learning rate, 0 < *d* ≤ 1 is the decay rate and the *H* and *WH* superscripts refer to Hebbian or Widrow-Hoff learning rules, respectively.

(1) $$\Delta w_{ij}^{H}=-d^{H}w_{ij}+k^{H}a_{i}a_{j}$$

(2) $$\Delta w_{ij}^{WH}=-d^{WH}w_{ij}+k^{WH}a_{i}(t_{j}-\sum_{i^{\prime }}\left(w_{i^{\prime }j}a_{i^{\prime }}\right))$$

First, we address the relationship between learning under Hebbian and Widrow-Hoff rules in an experiment conducted by McGuire et al. (2014). The parameter estimation task they used is effectively associative learning with a single cue, because the participants' task was simply to predict the value of a parameter during each trial. As only one stimulus exists in this paradigm, the *i* subscript becomes redundant, therefore we can say that *a*_*j*_ = *w*_*j*_ and both H and WH learning rules can be simplified to

(3) $$\Delta w_{j}^{H^{\prime }}=-d^{H^{\prime }}w_{j}+k^{H^{\prime }}a_{j}$$

and

(4) $$\Delta w_{j}^{WH^{\prime }}=-d^{WH^{\prime }}w_{j}+k^{WH^{\prime }}(t_{j}-w_{j}).$$

By equating $\Delta w_{j}^{H^{\prime }}=\Delta w_{j}^{WH^{\prime }}$, we can see that this statement is true whenever *k*^*H*′^ = *k*^*WH*′^ and *d*^*WH*′^ + *k*^*WH*′^ = *d*^*H*′^. This means that in parameter estimation tasks, learning according to the Widrow-Hoff rule can be perfectly mimicked by a Hebbian rule. Therefore, performance on this task cannot be used to argue for PE learning.

This proof cannot be extended to experiments with multiple cues (Čevora, 2017), such as the one by Gläscher et al. (2010). We therefore turn to computational simulations to investigate whether there is a correlation between Hebbian weight update and prediction error. In these simulations we look at whether PE correlates with weight update. Because fMRI observes entire populations of neurons, in contrast to single-cell recordings, we need to specify the variables of interest at the population level too.

We only consider the magnitude of the population weight change, |Δ*W*^*H*^|, because the fast decreases in synaptic strength are likely to require a similar amount of ATP as increases (Kandel, 2001; Collingridge et al., 2004) thus producing the same BOLD signal. Therefore the change associated with trial τ is:

(5) $$|\Delta W^{H}(\tau )|=\sum_{i}\sum_{j}|w_{ij}(\tau )-w_{ij}(\tau -1)|\;.$$

Likewise, we only consider the magnitude of the population PE, given that both positive and negative PE is likely to have metabolic consequences. We define this quantity, |*PE*|, as the sum of the absolute values of differences between predictions for each possible outcome, ||*a*_*j*_||, and the corresponding target values *t*_*j*_, on the current trial:

(6) $$|PE|=\sum_{j}|t_{j}-\|a_{j}\||\;,$$

where the prediction ||*a*_*j*_||:

(7) $$\begin{array}{cc} \|a_{j}\|=\frac{\sum_{i}a_{i}w_{ij}}{\sum_{j}\sum_{i^{\prime }}a_{i^{\prime }}w_{i^{\prime }j}} & \left(7\right) \end{array}$$

is a normalized activation vector as parameterizations of Hebbian learning do not produce predictions that can be interpreted directly as probabilities.

Another quantity of interest is resulting classification error after learning E. This is defined as a magnitude of difference between prediction and true (noiseless) outcome for each cue C, thus not only capturing how well the learning model can remember observations, but also how resilient it is to noise during learning:

(8) $$E=\sum_{C}\left(\sum_{j}t_{j}^{C}-\|a_{j}^{C}\|\right)\;.$$

Simulations were conducted for a number of possible experimental designs, for both categorical and continuous associative learning, with various degrees of stochasticity and various numbers of cues/outcomes. The simulations were run across the range of values for learning rate and weight decay that produce plausible learning dynamics (Figure 1). We recorded |Δ*W*^*H*^| and |*PE*| on each trial, and calculated the correlation between them.

**Figure 1:**
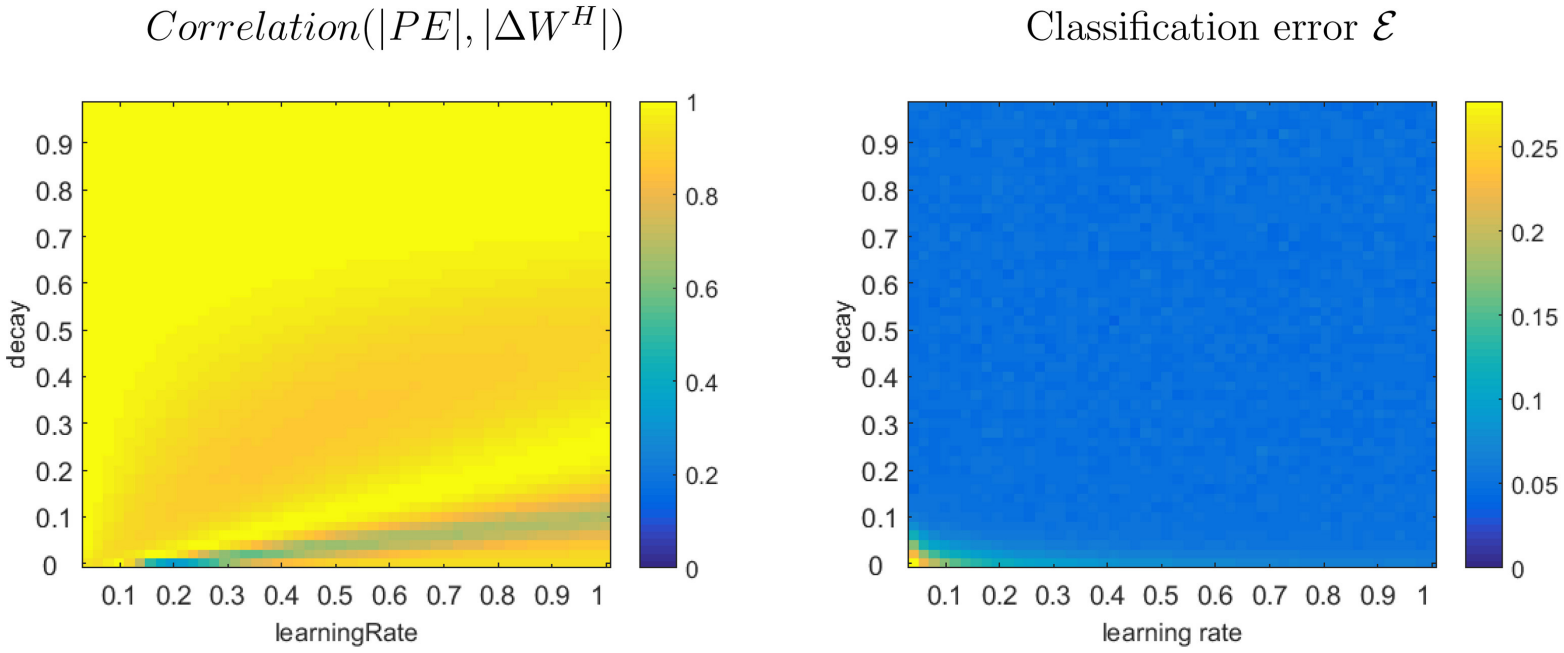
Left plot shows the correlation coefficient between |*PE*| and |Δ*W*^*H*^| as a function of learning rate and decay parameters of Hebbian learning during a quasi stochastic associative learning task. Right plot shows the average classification error E on the task after 50 learning trials. These particular plots reflect a learning situation where 4 cues are alternately associated with 4 distinct outcomes. 90% of the stimulus-outcomes pairs followed a particular bijective mapping, while the other stimulus-outcome pairs violated this mapping to introduce stochasticity.

The resulting correlations, plotted as a function of learning rate and weight decay, reveal that most of the parameter space results in strong correlations (Figure 1). Moreover, the classification error E is almost identical across the parameter space (except for a region in bottom left where both parameters are near zero), and therefore almost all parameter combinations are equally plausible for a real learner that tunes its learning parameters to the task. In other words, it is not the case that situations in which |*PE*| and |Δ*W*^*H*^| are highly correlated are non-optimal.

We conclude that, while there is convincing evidence that PE is computed by some neurons, the current evidence used to implicate this neural PE signal in learning has alternative explanations. There are a few fMRI studies that correlate brain activity with PE, a subset of which go further and link this to learning outcomes. However, due to the nature of BOLD signal measured by fMRI, the correlation with PE may not be a result of actual PE signaling, but rather a result of metabolic processes related to synaptic plasticity: Our computational modeling demonstrates that the magnitude of synaptic plasticity is highly correlated to PE, even when no PE computation takes place during learning. Note that this article has not addressed the role of PE at higher levels of analysis, such as the computational level (Marr, 1982), though one would expect such computation to still have a neural correlate. Further modeling and experimental paradigms are therefore needed to establish the basic form of learning rules underlying human associative learning.

## Author contributions

All work was carried out by JČ as a part of his Ph.D. under the supervision of RH.

### Conflict of interest statement

The authors declare that the research was conducted in the absence of any commercial or financial relationships that could be construed as a potential conflict of interest. The reviewer JK and handling Editor declared their shared affiliation, and the handling Editor states that the process nevertheless met the standards of a fair and objective review.
